# A green protocol for the electrochemical synthesis of a fluorescent dye with antibacterial activity from imipramine oxidation

**DOI:** 10.1038/s41598-022-08770-4

**Published:** 2022-03-22

**Authors:** Zahra Souri, Mahmood Masoudi Khoram, Davood Nematollahi, Mohammad Mazloum-Ardakani, Hojjat Alizadeh

**Affiliations:** 1 Department of Chemistry, Faculty of Science, Yazd University, Yazd, Iran; 2 Faculty of Chemistry, Bu-Ali Sina University, 65178-38683 Hamedan, Iran; 3 Rooyana Veterinary Laboratory, Saqqez, Kurdistan Iran

**Keywords:** Chemistry, Electrochemistry, Green chemistry

## Abstract

Electrochemical oxidation of imipramine (IMP) has been studied in aqueous solutions by cyclic voltammetry and controlled-potential coulometry techniques. Our voltammetric results show a complex behavior for oxidation of IMP at different pH values. In this study, we focused our attention on the electrochemical oxidation of IMP at a pH of about 5. Under these conditions, our results show that the oxidation of IMP leads to the formation of a unique dimer of IMP (DIMP). The structure of synthesized dimer is fully characterized by UV–visible, FTIR, ^1^H NMR, ^13^C NMR and mass spectrometry techniques. It seems that the first step in the oxidation of IMP is the cleavage of the alkyl group (formation of IMPH). After this, a domino oxidation-hydroxylation-dimerization-oxidation reaction, converts IMPH to (*E*)-10,10′,11,11′-tetrahydro-[2,2′-bidibenzo[b,f]azepinylidene]-1,1′(5*H*,5′*H*)-dione (DIMP). The synthesis of DIMP is performed in an aqueous solution under mild conditions, without the need for any catalyst or oxidant. Based on our electrochemical findings as well as the identification of the final product, a possible reaction mechanism for IMP oxidation has been proposed. Conjugated double bonds in the DIMP structure cause the compound to become colored with sufficient fluorescence activity (excitation wave-length 535 nm and emission wave-length 625 nm). Moreover, DIMP has been evaluated for in vitro antibacterial. The antibacterial tests indicated that DIMP showed good antibacterial performance against all examined gram-positive and gram-negative bacteria (*Staphylococcus aureus, Bacillus cereus, Escherichia coli* and *Shigella sonnei*).

## Introduction

Dibenzazepines, as electron-rich compounds, are one of the most important organic substances that have received much attention in academic and technological research^1–3^. These compounds have been known since 1899 when Tille and Holzinger synthesized 10,11-dihydrodibenzo[b,f]azpine^4,5^. These compounds can serve as photoactive and electroactive materials in molecular electronics^6^, electrogenerated chemiluminescence^7^, efficient nonlinear optical materials^8^, dye-sensitized solar cells (DSSCs)^9–11^, and organic light-emitting diodes (OLEDs)^12^. In addition, dibenzazepines having N–H bond have attracted much attention because compounds with Ar_2_NH structure have always been the central structure in many drugs^13,14^. Due to the great interest in dibenzazepine derivatives, many synthetic procedures have been developed for such compounds including, the dehydrogenation of iminobibenzyls^15,16^, the rearrangement of arylindoles^17^ and metal catalyzed synthesis^18,19^. The above methods have several disadvantages such as: using transition metal and toxic chemical reagent, require prefunctionalized substrates, produce undesired toxic side products, heavy metal waste, high temperature, undesirable solvent, functional group-intolerant conditions, low yields and tedious workup^18,20–22^. Besides these methods, electrochemical methods were also reported for the synthesis of dibenzazepine derivatives^23–26^. Electrochemical methods have a wide range of applications in the synthesis of electroactive compounds due to their excellent performance and no need for toxic chemical reagents or expensive noble metals^25,27–39^. Moreover, the discovery of new electrode materials, the development of multifunctional practical straightforward equipment, along with bio-renewable solvents will provide in the future robust, general and scale-up electrosynthetical processes compared to traditional methods^40^. In this regard, this work has been conducted with the aim of elucidating the electrochemical oxidation of imipramine (IMP) and creating a new oxidation pathway for IMP. Imipramine is a tricyclic antidepressant used for the treatment of physiological retardation depression, bipolar disorder, dysthymia, hyperactivity disorder^23,41^. The oxidation mechanism of imipramine has been investigated in only a few studies^41–45^. This encourages us to do a more complete study on the oxidation pathway of IMP. On this subject, the electrocatalytic degradation of imipramine with fluorine-doped β-PbO_2_ electrode^46^ and the electrochemical polymerization of imipramine at pH 1.0^46^ have recently been studied by us. In order to complete the previous studies^46,47^, in this work, we investigate the electrochemical oxidation of imipramine in acetate buffer (pH 5.0) and we managed to synthesize a unique dimer (DIMP) from this compound. This electroorganic synthesis is performed in one step using efficient and ecofriendly methods in high yield and purity without toxic reagents and solvents at a carbon electrode in an undivided cell. The results show that the first step in the hydroxylation/dimerization of IMP is the oxidative dealkylation process. At this point, the alkyl chain separates from the IMP. After this step, a series of reactions, including oxidation, hydroxylation, dimerization and oxidation, lead to the synthesis of (*E*)-10,10′,11,11′-tetrahydro-[2,2′-bidibenzo[b,f] azepinylidene]-1,1′(5*H*,5′*H*)-dione (DIMP) as a fluorescent dye with antibacterial activity.

## Results and discussion

### Mechanistic studies

**Figure 1:**
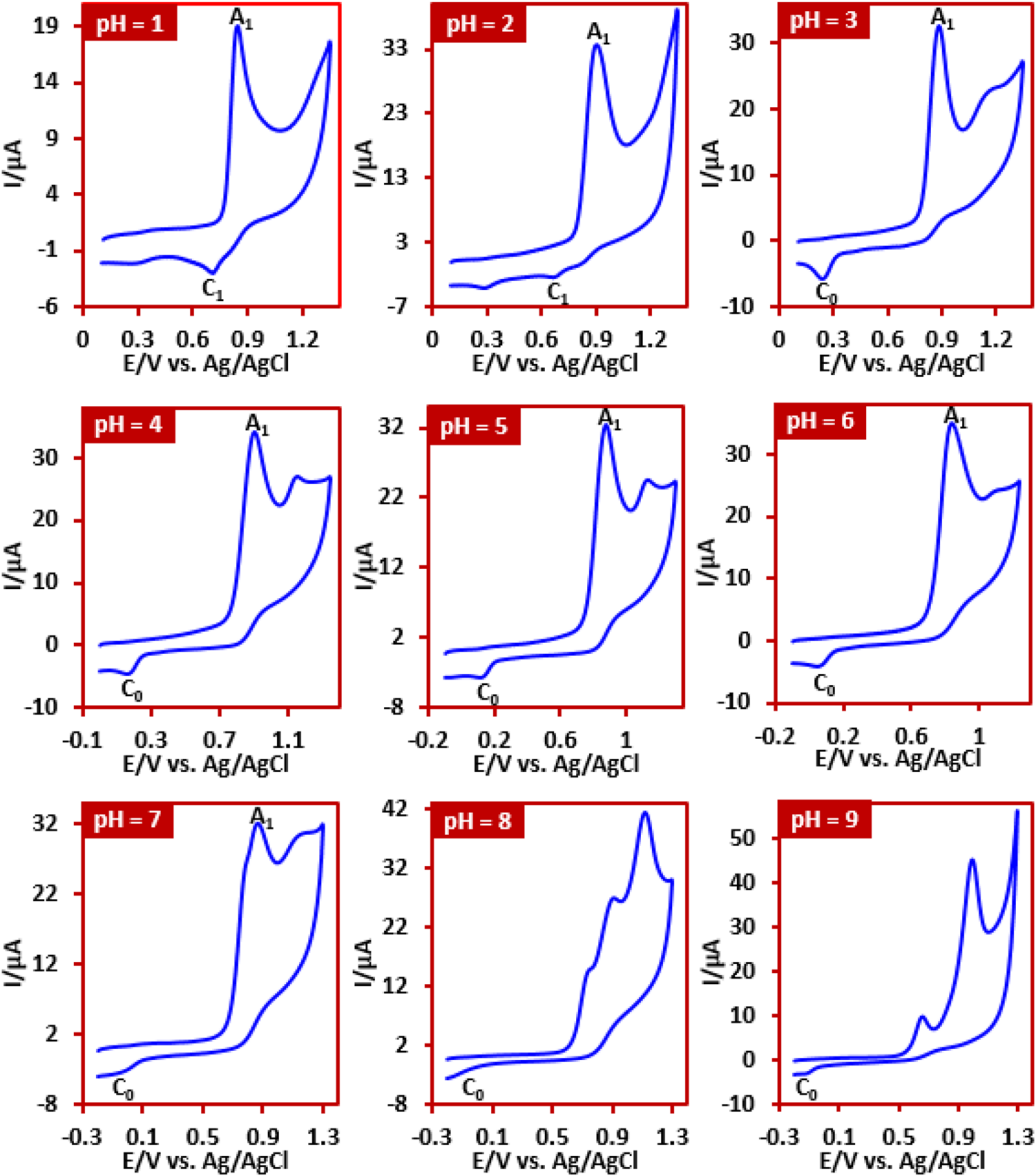
Cyclic voltammograms of IMP (1.0 mM) at glassy carbon electrode in aqueous solutions at different pH values. Scan rate: 100 mV s^−1^, at room temperature.

Cyclic voltammetry was used to study the redox behavior, pH-dependent properties and electron transfer mechanism of IMP in aqueous solution. The pH dependent behavior of IMP was investigated by cyclic voltammetry. Figure 1 shows the cyclic voltammograms of IMP at different pH values. As can be seen, the IMP exhibits complex and different behaviors. For example, while peak C_1_ is present at pH values less than 2, it is not seen at higher pH values. Or peak C_0_ is seen in the pH range 3–6 and is gradually removed as the pH increases. On the other hand, while voltammograms show one or two anodic peaks up to pH 6, the number of anodic peaks increases to three as the pH increases further. The fundamental change in the electrochemical behavior of IMP in alkaline media may be due to the participation of IMP in hydroxylation and/or hydrolysis reactions. These results indicate high complexity in IMP oxidation. In previous studies, we have been able to identify some oxidation pathways of this compound^46,47^. Based on our previous data, peak A_1_ is attributed to the one-electron oxidation of IMP to the corresponding radical cation and peak C_1_ is related to the reduction of radical cation to IMP^46,47^. In this work, we pay attention to the behavior of IMP in the pH range 3–6.

**Figure 2:**
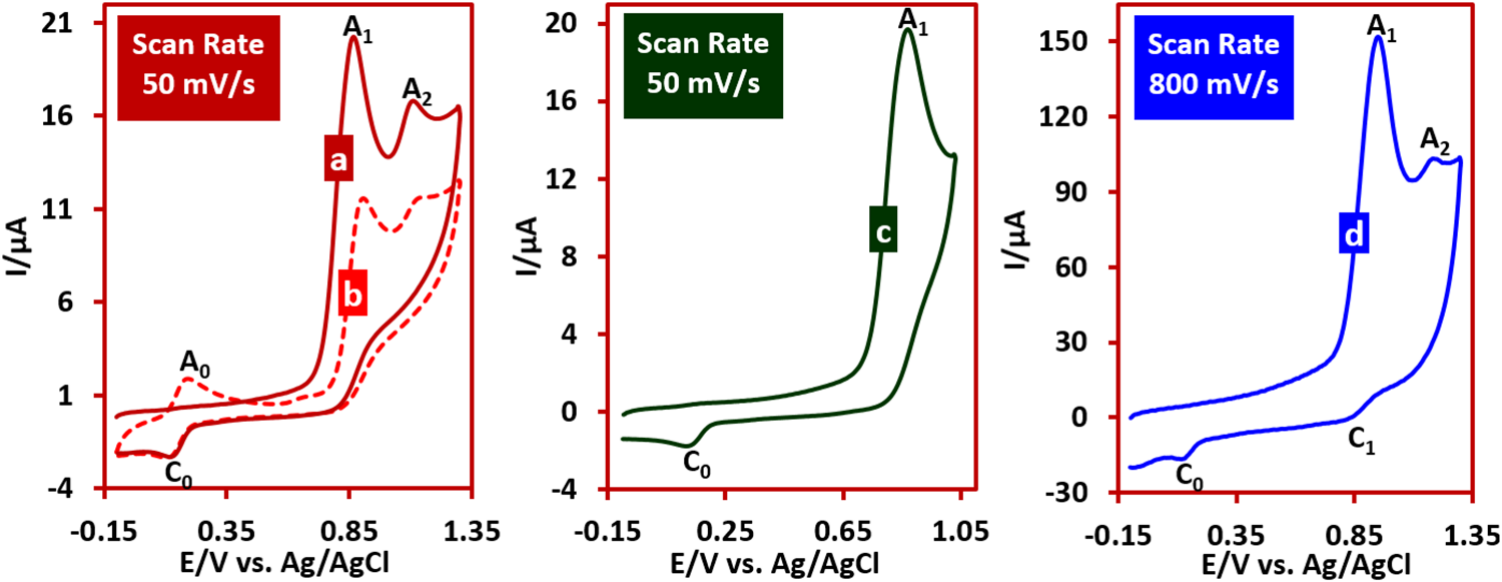
Curves (a, b) First and second cyclic voltammograms of IMP (1.0 mM) at glassy carbon electrode in water (acetate buffer, *c* = 0.2 M, pH 5.0), at room temperature. Curve © is similar to curve a, but with a more limited potential range. Curve (d) is similar to curve a, but at scan rate of 800 mV s^−1^.

Figure 2, curves a and b shows the first and second cyclic voltammograms of IMP in the aqueous solution (pH 5.0) at scan rate of 50 mV s^−1^. As can be seen, in the first scan, two anodic peaks A_1_ and A_2_ are observed over the whole potential range. When the potential is switched to negative potentials, a new reduction peak (C_0_) appears at a potential of 0.13 V versus Ag/AgCl and in the second cycle, a new anodic peak (A_0_) (counterpart of C_0_ peak) appears with *E*_p_ of 0.19 V versus Ag/AgCl. Figure 2, curve c shows the cyclic voltammogram of IMP over the limited potential range up to 1.0 V versus Ag/AgCl. The presence of peak C_0_ in the voltammogram in these conditions indicates its independence from peak A_2_. Increasing the scan rate in these situations has three important consequences (Fig. 2, curve d). First, decrease the anodic peak current ratio (*I*_pA2_/*I*_pA1_). Second, decrease the cathodic-to anodic peak current ratio (*I*_pC0_/*I*_pA1_) and third, appearance of the peak C_1_.

**Figure 3:**
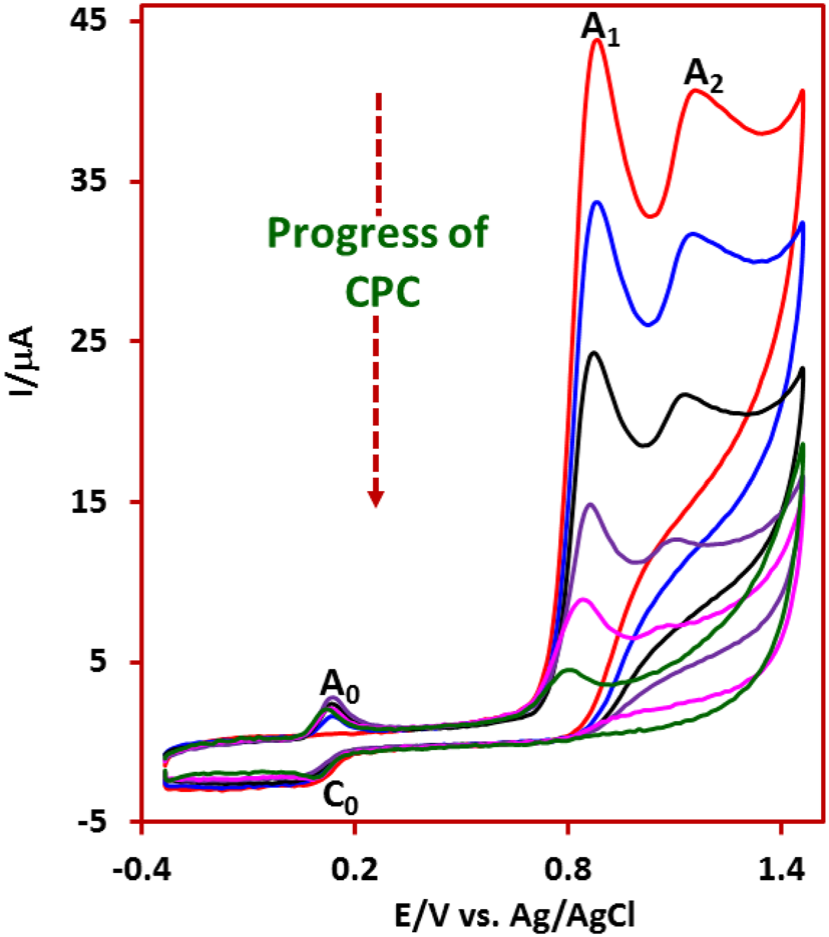
Cyclic voltammograms of IMP (0.25 mmol) during controlled-potential coulometry at + 0.85 V versus Ag/AgCl in water (acetate buffer, *c* = 0.2 M, pH 5.0). Scan rate: 50 mV s^−1^, at room temperature.

In order to better understand the oxidation reaction mechanism at pH 5, here we performed a controlled-potential coulometry (CPC) experiment to determine the number of transferred electrons and recorded the cyclic voltammograms of the solution during the coulometry. For this purpose, CPC was carried out in aqueous solution (80 ml buffer, pH 5.0) containing 0.25 mmol of IMP at the potential of 0.85 V versus Ag/AgCl. Figure 3, shows the cyclic voltammograms of IMP during controlled-potential coulometry. As can be seen, *I*_pA1_ and *I*_pA2_ decrease with time (electricity consumption), while *I*_pA0_ and *I*_pC0_ increase slightly. Also, the number of electrons exchanged in this experiment was found to be slightly more than 6 electrons.

**Figure 4:**
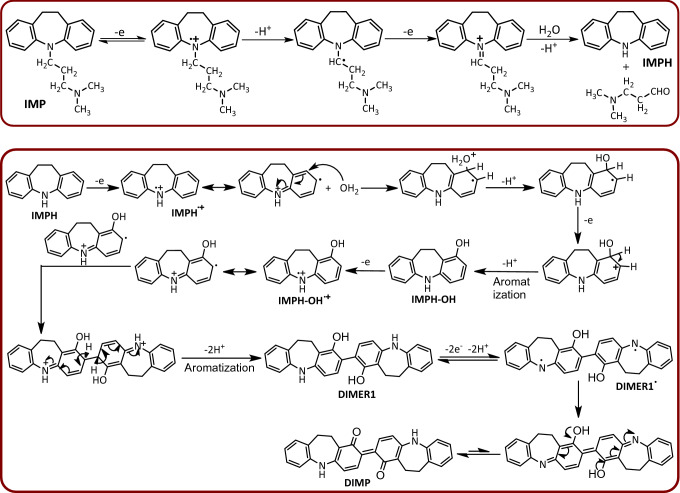
Electrochemical synthesis of DIMP by a domino dealkylation-oxidation-hydroxylation-dimerization-oxidation reaction.

According to these results together with the spectroscopic data of the isolated product as well as our previous studies^46,47^, we proposed the following mechanism for the electrochemical oxidation of IMP at the pH 5 (Fig. 4). Since the spectroscopic data of the synthetic product show that the alkyl group is not involved the final product, it seems that the first step in the oxidation of IMP is the cleavage of the alkyl group from IMP (formation of IMPH), during an oxidative dealkylation reaction^48^. In the next step, IMPH is oxidized to the corresponding radical cation via a one-electron-transfer process. This compound is converted to IMPH-OH after hydroxylation (due to water attack) and then aromatization. In the next step, the IMPH-OH is converted to the corresponding radical cation by a one electron transfer process (IMPH-OH^•+^). The reaction of two radical cations of IMPH-OH together, followed by aromatization, forms the corresponding dimer (DIMER1). Finally, a two-electron oxidation process, together with a rearrangement, converts DIMER1 to the final product (DIMP).

**Figure 5:**
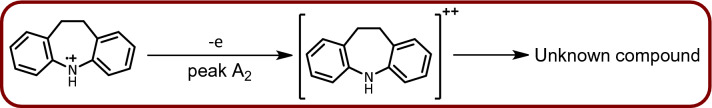
Proposed reaction for generation of peak A_2_.

Based on the available results, peak A_1_ and its cathodic counterpart (C_1_) (see Fig. 1) are correspond to one-electron oxidation of IMP to IMP^•+^ and vice versa. In this regard, due to the removal of peak A_2_ at high scan rates, this peak is related to the over-oxidation of IMPH^•+^ to IMPH^++^ (Fig. 5).

**Figure 6:**
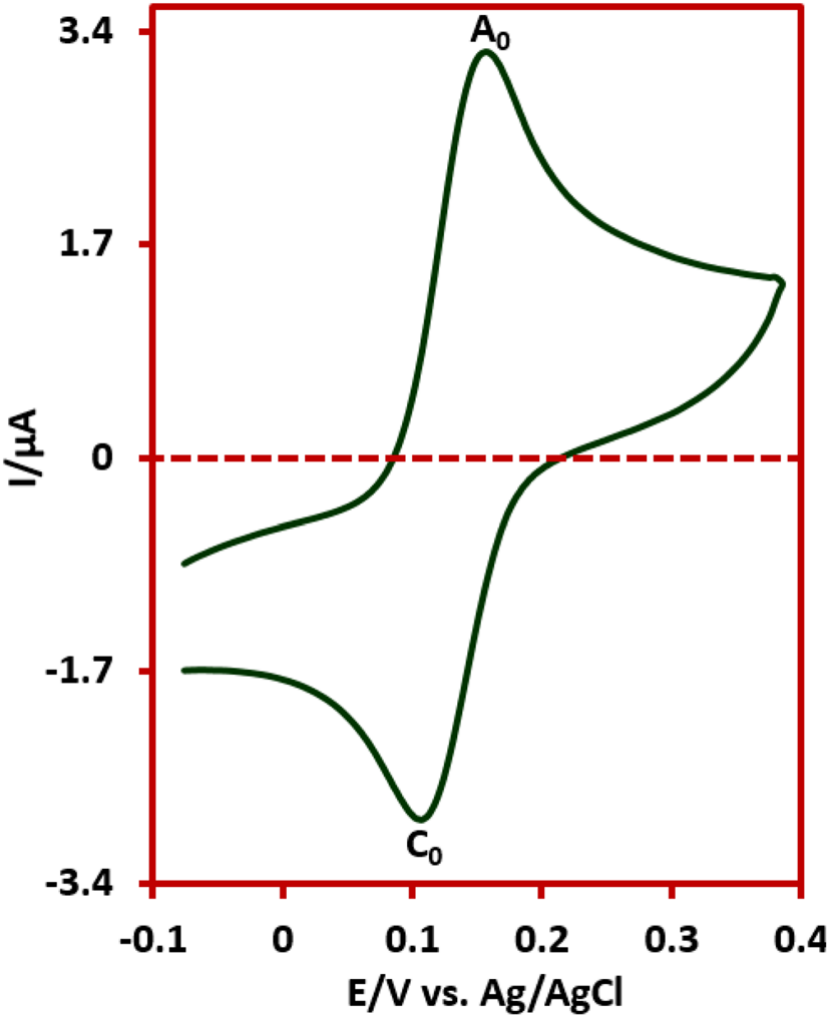
Cyclic voltammogram of saturated solution of synthe sized product (DIMP) in coulometric conditions, at room temperature.

**Figure 7:**
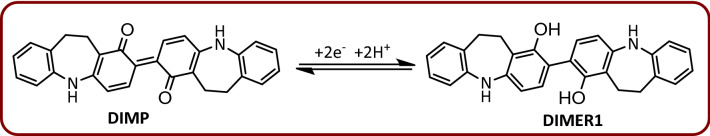
The redox behavior of A_0_/C_0_ peaks.

In order to determine the species causing peaks A_0_ and C_0_, the cyclic voltamogram of the isolated product (DIMP) is shown in Fig. 6. The cyclic voltammogram shows a pair of anodic and cathodic peaks at 0.16 and 0.11 V versus Ag/AgCl, respectively, which are compatible with the potential of peaks A_0_ and C_0_. Accordingly, the molecules that create peaks A_0_ and C_0_ and the redox behavior of them are shown in Fig. 7.

The second point that can be understood from this cyclic voltammogram is the presence of cathodic currents at the beginning of the potential scan. These currents are related to the reduction of the compound present at the electrode surface before the compound is oxidized at the potential of peak A_1_. In other words, these currents indicate that the product is in oxide form. These results are consistent with the structure of the DIMP which is in its oxidized form.

**Figure 8:**
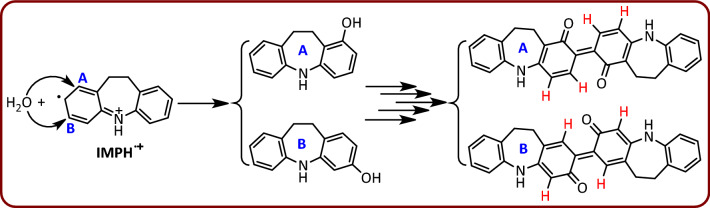
Possible structures due to the increase of water to different positions of the IMPH^•+^ molecule.

An important point to note about hydroxylation of IMPH^•+^ is the location of the hydroxyl group in IMPH-OH molecule^49,50^. IMPH^•+^ can be attacked by water from two places A and B and become two different products according to Fig. 4 (Fig. 8). In the structures shown in Fig. 8, the hydrogens of the quinone rings have different positions relative to each other. In structure A, the hydrogens of the quinone rings are in *ortho* positions, while in structure B, the hydrogens of the quinone rings are in *para* positions. We confirm the formation of structure A according to the results obtained from the NMR spectrum. The ^1^H NMR spectrum of the reaction product (DIMP), shows clearly two doublet peaks with *J* = 10 and 8 Hz at δ 6.64 and 7.64 ppm, respectively, showing coupling to the two *ortho* protons. These results are consistent with structure A.

**Figure 9:**
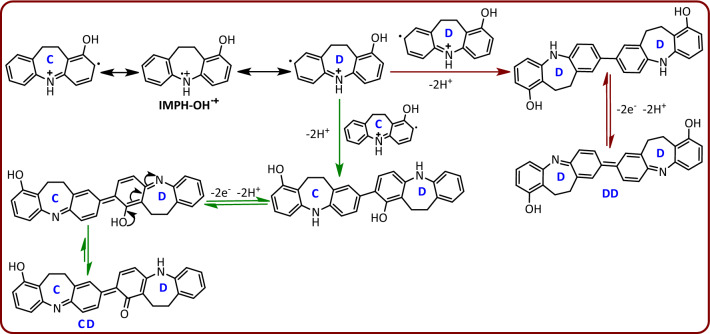
Other mechanisms for the dimerization of IMPH-OH.

In addition, it is possible that the rearrangement of IMPH-OH^•+^ is preceded by different mechanisms and leads to the formation of a different products (DD and CD), as shown in Fig. 9. But we reject these mechanisms because of the results obtained from NMR spectra. As reported in the experimental section, the carbon NMR spectrum of the synthesized compound has a peak at 187.8 ppm, which corresponds to carbonyl groups, while the product, DD, shown in Fig. 9 has no carbonyl group. This finding causes us to reject the formation of DD product. On the other hand, the structure of the CD compound shows that this molecule is an asymmetric molecule, so that in its proton and carbon spectra, the number of peaks and their patterns are completely different from that of the synthesized product (DIMP). These findings rule out the formation of CD molecule as a final product.

### UV–visible and fluorescence characteristics of DIMP

**Figure 10:**
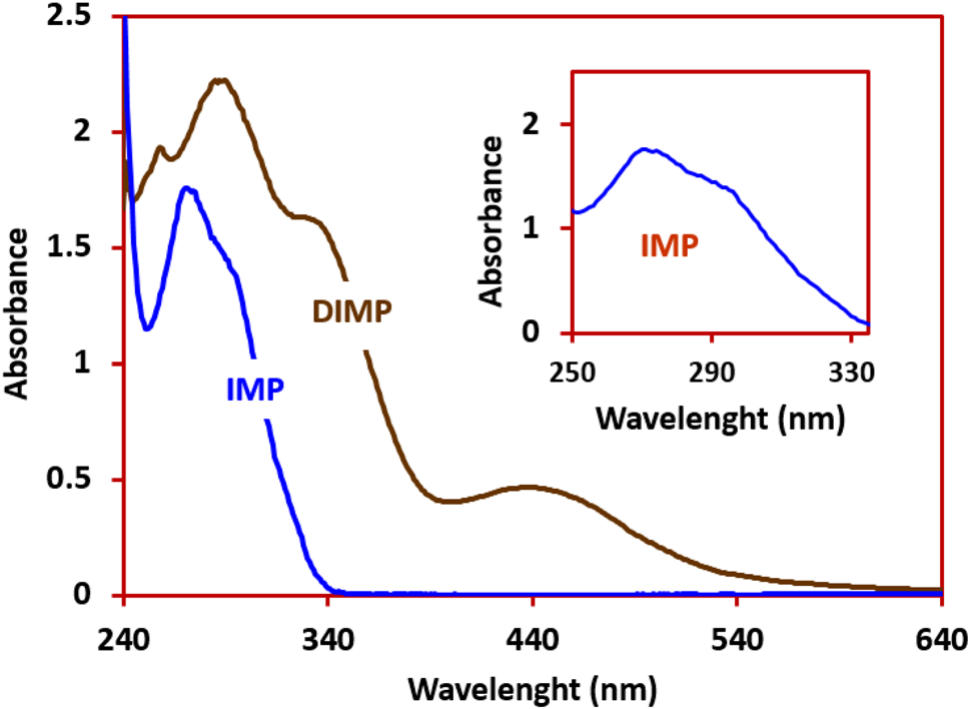
UV–visible spectra of IMP (0.5 mM) and DIMP (0.5 mM) in CHCl_3_. Inset: expanded IMP.

In this part, spectrophotometric techniques were used to characterization of the ground state and the singlet excited state of synthesized product (DIMP) by means of absorption and fluorescence methods. Figure 10 shows the UV–visible spectrum of 0.5 mM IMP and DIMP in chloroform. As can be seen, the absorption spectrum of IMP shows two bands at 270 and 295 nm were attributed to π → π* transitions associated with the aromatic rings^9,51,52^ (Fig. 10, inset). A red shift was observed when the UV–visible spectrum has been carried out from DIMP, which can be related to the absence of the *N*-alkyl chain which has been replaced by hydrogen during electrochemical oxidation^53^. This result indicates that the alkyl chain-substituent affects the electronic transitions of the chromophore^53^. Also, the broad band from 400 to 520 nm with its maximum centered at 440 nm, is due to the conjugated double bonds^51^. The synthesized compound (DIMP) is colored (reddish yellow) and therefore may be used as a dye^54–56^. Based on this, the color quality of this compound has been evaluated and approved in the quality control laboratory of Alvan Sabet Company.

**Figure 11:**
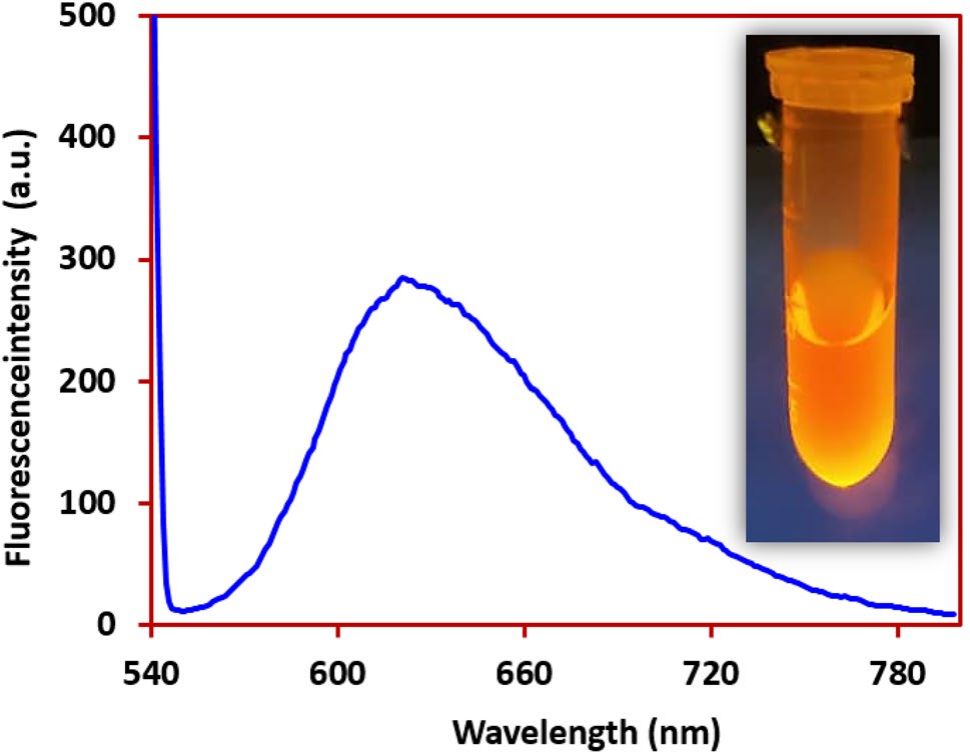
Fluorescent spectrum of DIMP and the photograph of DIMP (0.5 mM) using an ultraviolet lamp in CHCl_3_ solvent measured under excitation of 535 nm.

Figure 11 shows the fluorescence spectrum of 0.5 mM DIMP in chloroform. For this experiment the fluorescence was monitored at an excitation wavelength of 535 nm at a 90° angle relative to the excitation light. Under these conditions, the emission wavelength of DIMP was found to be 625 nm. The conjugated bands in the structure of DIMP (Fig. 4) caused to emergence of fluorescence properties in this compound.

### Antibacterial susceptibility

**Figure 12:**
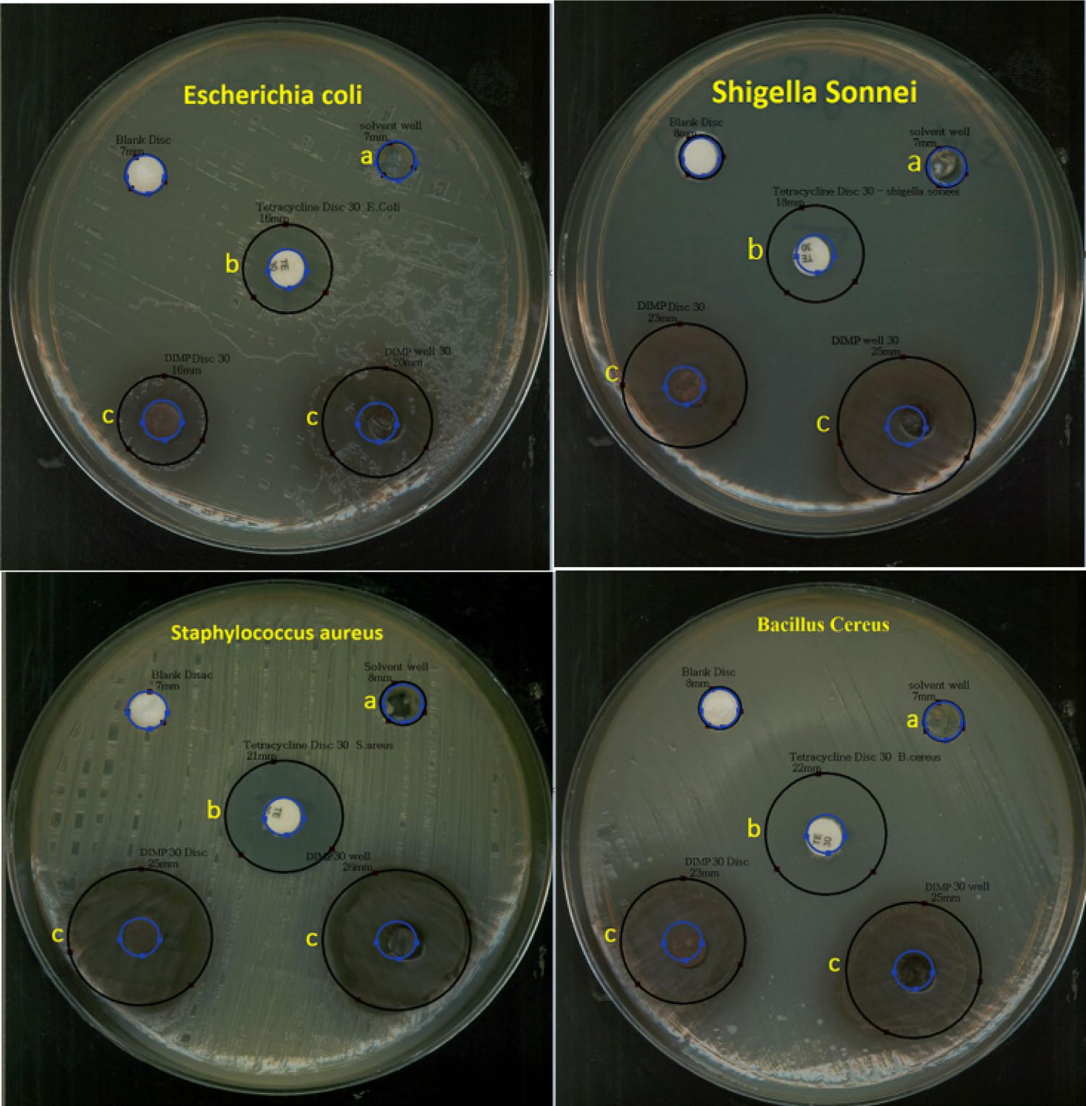
Inhibition zone diameters (mm) obtained of *Bacillus* cereus, *Staphylococcus* aureus, *Escherichia* coli and *Shigella* sonnei in disc diffusion test for 30 mg ml^−1^ of (a) solvent, (b) tetracycline and (c) DIMP.

The synthesized product DIMP was tested to evaluate the antibacterial activity. The effect of DIMP (30 mg ml^−1^) on the four strains was assayed by agar well diffusion method and further confirmed by disk diffusion method. Four bacterial: *Bacillus* cereus (ATCC 14759), *Staphylococcus* aureus (ATCC 29213), *Escherichia* coli (ATCC 25922) and *Shigella* sonei (ATCC 9290) used in our study (Fig. 12). Antobiogram test showed that the all gram positive and all gram negative bacteria (tested in this research) was sensitive to DIMP. For determination of minimum inhibition concentrations (MIC) of the DIMP to inhibit the microorganisms more studies are necessary and microdilution method is recommended. The result of antibacterial activity of the DIMP compound is summarized in Table 1. These properties introduce DIMP as a fluorescent dye with antibacterial activity.

**Table 1:** Antibacterial activity of DIMP by agar well-diffusion method.

Name of the organisms	Solvent well	Blank disk	Tetracycline disc 30 µg	Well 30 µg	Disc 30 µg	Activity
*Bacillus* cereus	−	−	22	25	23	(+)
*Staphylococcus* aureus	−	−	21	26	25	(+)
*Escherichia* coli	−	−	16	20	16	(+)
*Shigella* sonnei	−	−	18	25	23	(+)
*Bacillus* cereus ATCC 14759					Gram positive	
*Staphylococcus* aureus ATCC 29213					Gram positive	
*Escherichia* coli ATCC 25922					Gram negative	
*Shigella* sonnei ATTC 9290					Gram negative	

(+) Positive activity; (−) negative activity; - no inhibition, tetracycline = standard drug; Solvent well = chloroform + Tween20.

## Conclusions

In this study, the electrochemical synthesis of DIMP is reported as a new derivative of dibenzazepine in good yield and purity via C–C bond formation. To achieve this goal, the electrochemical behavior of IMP at different temperatures was first investigated and it was found that the best pH value for the synthesis of DIMP is 5. The results show that under these conditions, the oxidation of IMP proceeds through a complex path. It seems that the first step in the synthesis of DIMP is oxidative dealkylation of IMP. After this step, a series of reactions, including oxidation, hydroxylation, dimerization and oxidation, convert the dealkylated IMP to (*E*)-10,10′,11,11′-tetrahydro-[2,2′-bidibenzo[b,f] azepinylidene]-1,1′(5*H*,5′)-dione (DIMP). The synthesis of DIMP is carried out in the aqueous solution under mild conditions in one-pot without use of any toxic chemicals or organic solvents, with a very simple procedure for separation and purification. The structure of DIMP is fully characterized by UV-visible, FTIR, ^1^H NMR, ^13^C NMR and mass spectrometry techniques. Conjugated double bonds in the structure of DIMP cause the compound to become colored with sufficient fluorescence activity. In addition, the antibacterial tests indicated that DIMP showed good antibacterial performance against all examined gram-positive and gram-negative bacteria (*Staphylococcus aureus, Bacillus cereus, Escherichia coli* and *Shigella sonnei*). These properties make the DIMP known as a unique fluorescent dye with antibacterial properties.

### Reagents and apparatus

Cyclic voltammetry, controlled potential coulometry and macroscale electrolysis were performed using an Autolab model PGSTAT 20 potentiostat/galvanostat. Absorption spectra was taken with a Lambda 25 UV–Vis spectrophotometer. Fluorescence spectra was determined with a Varian spectrofluorometer. Both emission and excitation bands were set at 5 nm. The working electrode used in the voltammetry experiments was a glassy carbon disc (1.8 mm diameter) and a platinum wire was used as the counter electrode. The working electrode used in controlled-potential coulometry and preparative electrolysis was an assembly of four ordinary soft carbon rods (6 mm diameter and 4 cm length), while the counter electrode was a stainless-steel cylinder. The working electrode potentials were measured versus Ag/AgCl (all electrodes from AZAR Electrodes). Imipramine hydrochloride, 3-(10,11-dihydro-5*H*-dibenzo[b,f]azepin-5-yl)-*N,N*-dimethylpropan-1-aminium chloride (C_19_H_23_N_2_^+^Cl^−^) (Temad company, Iran), (MW = 280.407 g mol^−1^ and M. p: 174–175 °C) as the active substance of imipramine was reagent-grade. Other chemicals were obtained from commercial source and used without further purification.

### Electrochemical synthesis of DIMP

Controlled-potential electrolysis was used as a preparative method for the synthesis of DIMP. Achieving this goal, in an undivided cell equipped with carbon anode and stainless steel cathode, an aqueous solution (80 ml acetate buffer, pH 5.0) containing IMP (0.25 mmol) was electrolyzed at potential of 0.85 V versus Ag/AgCl. Electrolysis was discontinued when the current dropped to 5% of its initial value. Due to the fouling of electrode surface, the electrolysis is sometimes stopped and the carbon anode was washed with acetone in order to reactivate it. At the end of the electrolysis the cell was allowed to room temperature overnight. The precipitated solid was collected by filtration and washed several times with water. The product was purified by thin layer chromatography (ethyl acetate/*n*-hexane 50/50 v/v). After purification, product was characterized by UV–visible, IR, ^1^H-NMR, ^13^C-NMR, MS and melting point (M. p). Isolated yield: 75%. M. p: 138–139 °C, ^1^H NMR (400 MHz, CHCl_3_-*d*) *δ* 2.85 (t, 4H, CH_2_ aliphatic), 2.96 (t, 4H, CH_2_ aliphatic), 6.31 (s, 2H), 6.64 (d, 2H, *J* = 10.0 Hz), 7.23 (d, 2H, *J* = 7.0 Hz), 7.29–7.31 (m, 4H), 7.40 (t, 2H), 7.64 (d, 2H, *J* = 8.0 Hz). ^13^C NMR (100 MHz, CHCl_3_-*d*) *δ* 31.5, 34.8, 127.9, 128.8, 129.7, 130.2, 130.5, 134.1, 135.0, 145.0, 146.0, 146.5, 155.1, 187.8. IR (KBr, cm^−1^): 2924, 2855, 1741, 1642, 1615, 1593, 1489, 1350, 1293, 1155, 1089, 899, 817, 753, 668, 583. MS (EI, 70 eV): m/z (relative intensity): 420 (M + 2H, 0.1), 243 (13.8), 209 (66.2), 180 (100), 152 (12.7), 128 (8.4), 109 (2.3), 89 (19.3), 63 (13.7), 43 (7.7).

### Antibacterial studies

Agar well-diffusion method and disc diffusion method was followed to determine the antimicrobial activity of DIMP^57,58^. This material is melted in chloroform + Tween 20. The effect of DIMP 30 mg/ml on the four strains were assayed by agar well diffusion method and further confirmed by dsc diffusion method. Four bacterial ATCC strains (*Bacillus* cereus, *Staphylococcus* aureus, *Escherichia* coli, *Shigella* sonnei) used in this study. The bacterial strains were first incubated in brain heart infusion broth (BHI)^59,60^. After overnight incubation at 37 °C, 10 μL of the broth medium was streaked onto nutrient agar and then incubate for 24 h in the same condition. Then concentration of bacterial, was balanced with a 0.5 McFarland standard. In agar well-diffusion method, Muller hinton agar (MHA) plates were inoculated with bacteria and punched with a glass capillary to create well then filled with 30 mg each the samples , Solvent well (chloroform + Tween20), blank disc and standard drug (tetracycline). The plates were incubated at 37 °C for 24 h. In disc diffusion method, Muller hinton agar (MHA) plates were inoculated with bacteria then used discs prepared with 30 mg each the samples, Solvent disc (chloroform + Tween20), blank disc and standard drug disc (tetracycline). The plates were incubated at 37 °C for 24 h. Finally, the inhibition zone surrounding the wells and disc were measured to evaluate of antibacterial activity.

## Supplementary Information

Supplementary Information.
