# Supplementary material for: A green protocol for the electrochemical synthesis of a fluorescent dye with antibacterial activity from imipramine oxidation
Source: Sci Rep. 2022 Mar 22;12:4921. doi: 10.1038/s41598-022-08770-4 (PMC8941072; doi:10.1038/s41598-022-08770-4)
Supplement: Supplementary file 1 — Supplementary Information. [file 41598_2022_8770_MOESM1_ESM.docx]

**Supplementary data**

**A Green Protocol for the Electrochemical Synthesis of a Fluorescent Dye with Antibacterial Activity from Imipramine Oxidation**

Zahra Souri^1^, Mahmood Masoudi Khoram^2^, Davood Nematollahi^*2^, Mohammad Mazloum-Ardakani^1^, & Hojjat Alizadeh^3^

^1^Department of Chemistry, Faculty of Science, Yazd University, Yazd, Iran. ^2^Faculty of Chemistry, Bu-Ali Sina University, Hamedan, Iran. Zip Code 65178-38683. ^3^Rooyana Veterinary Laboratory, Saqqez, Kurdistan, Iran. E-mail: nemat@basu.ac.ir. Fax: 0098 - 813- 8257407, Tel: 0098 - 813- 8282807

**Table of contents**

| **No.** |  | **Page** |
| --- | --- | --- |
| **1** | IR spectrum of **IMP** | **S3** |
| **2** | IR spectrum of **DIMP** | **S4** |
| **3** | ^1^H NMR spectrum of **DIMP** | **S5** |
| **4** | Expanded ^1^H NMR spectrum of **DIMP** | **S6** |
| **5** | ^13^C NMR spectrum of **DIMP** | **S7** |
| **6** | MS spectrum of **DIMP** | **S8** |

FTIR spectrum of **IMP**


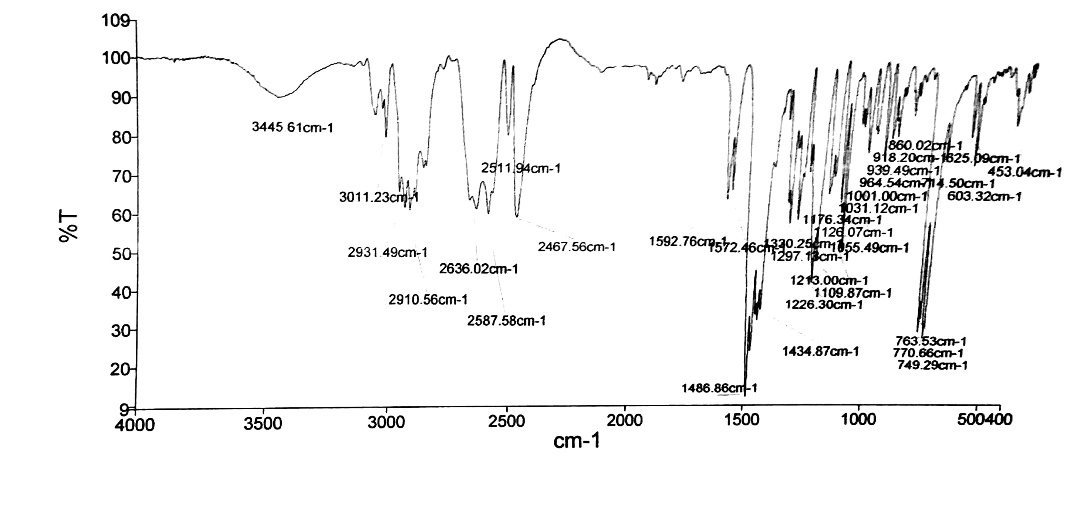


**Figure S1**. FTIR spectrum of **IMP.**

IR spectrum of **DIMP**


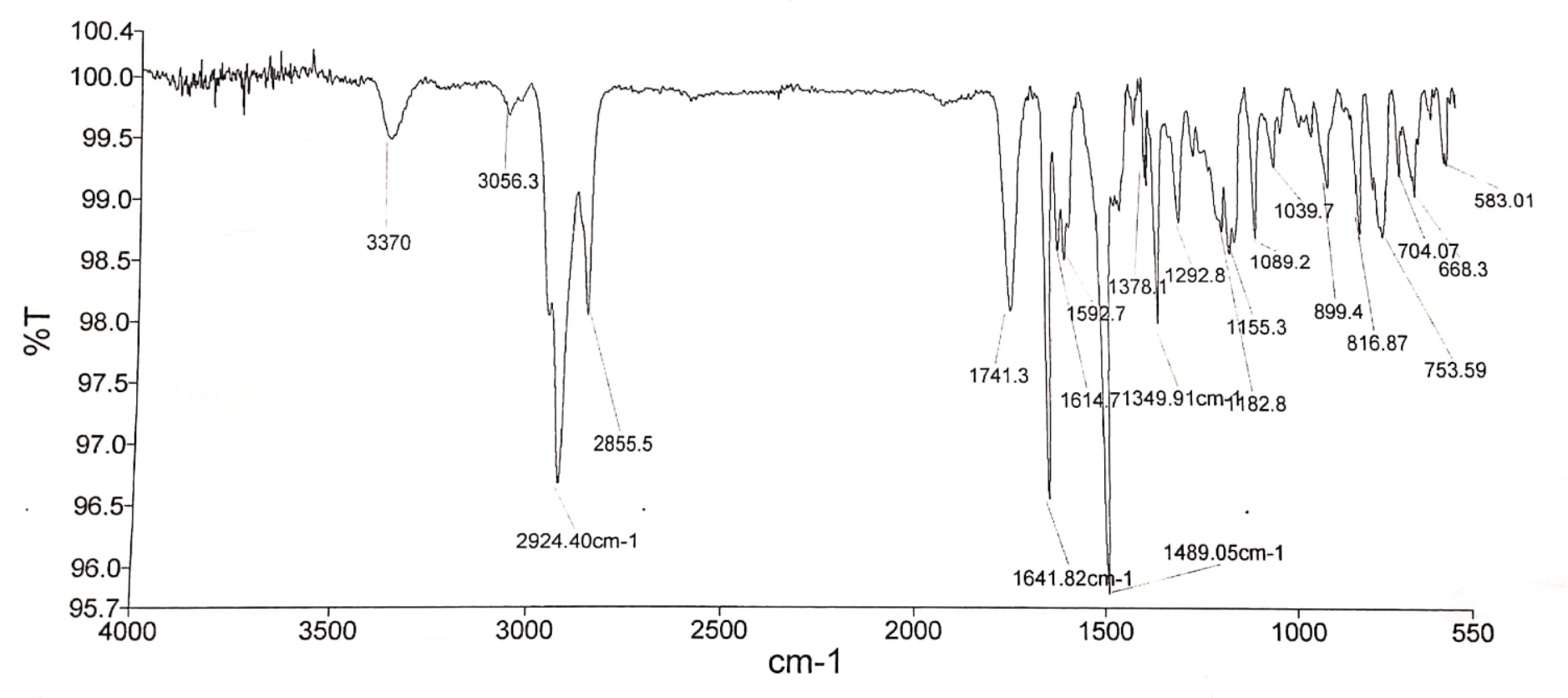


**Figure S2**. FTIR spectrum of **DIMP**.

^1^H NMR spectrum of **DIMP**


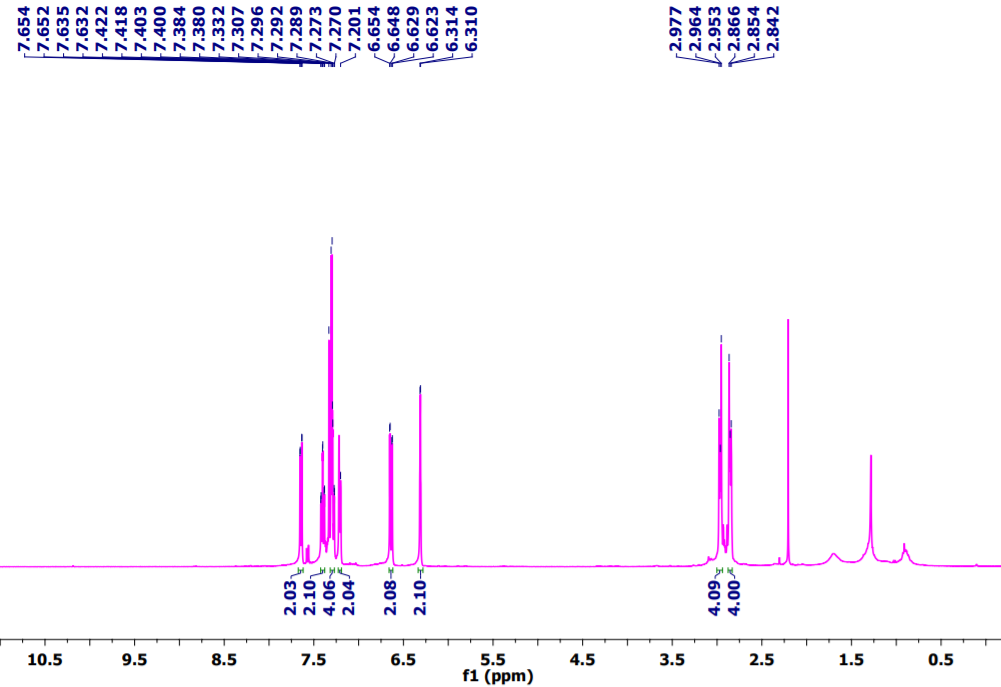


**Figure S3**. ^1^H NMR spectrum of **DIMP.**

Expanded ^1^H NMR spectrum of **DIMP**


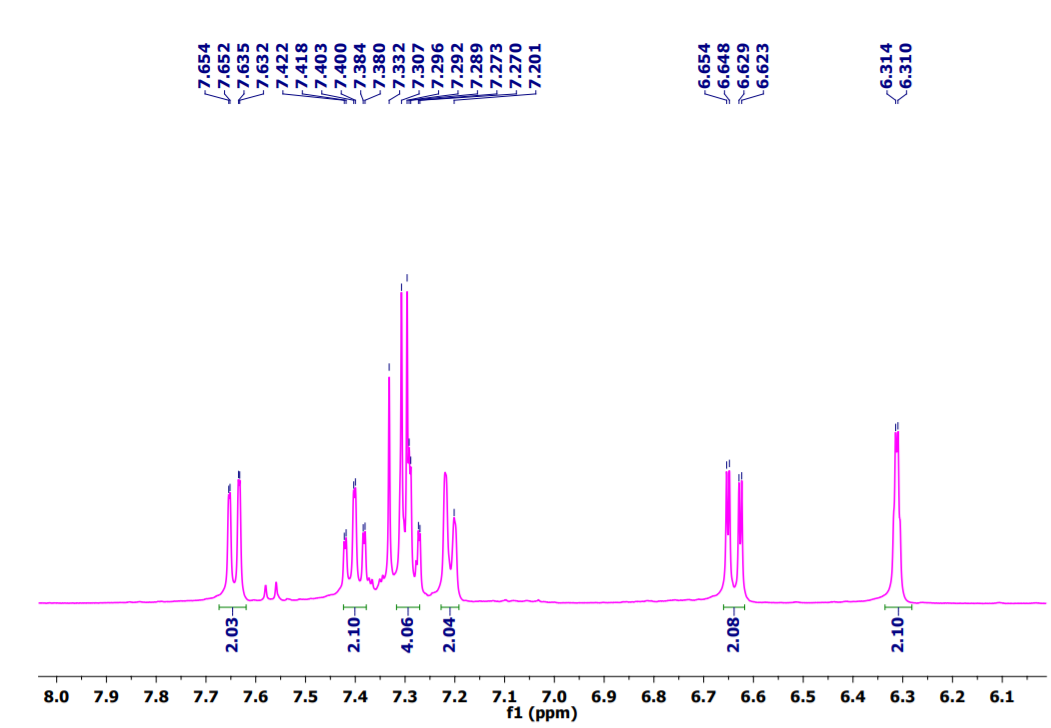


**Figure S4**. Expanded ^1^H NMR spectrum of **DIMP.**

^13^C NMR spectrum of **DIMP**


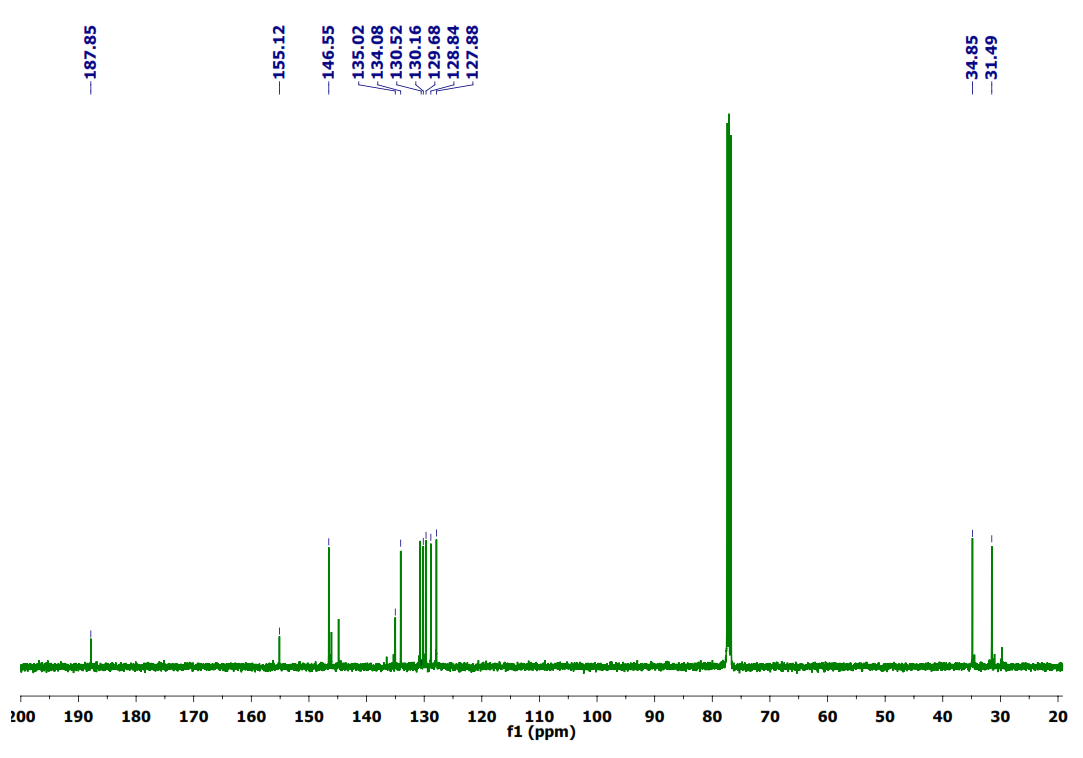


**Figure S5**. ^13^C NMR spectrum of **DIMP.**

MS spectrum of **DIMP**


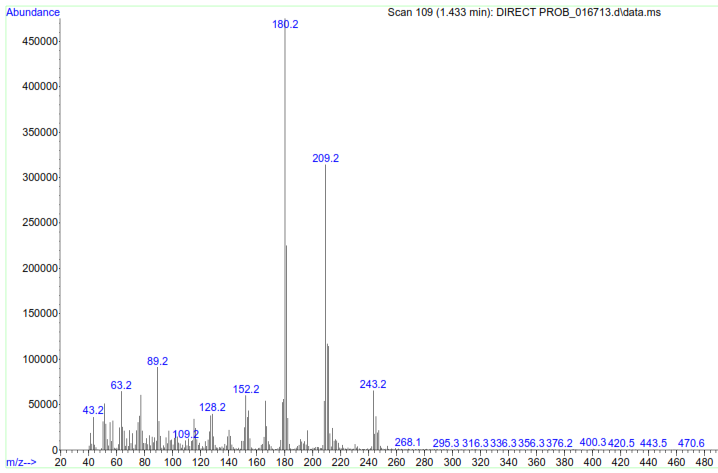


**Figure S6**. MS spectrum of **DIMP.**
